# The importance of institutional quality: Reviewing the relevance of Indonesia’s Omnibus Law on national competitiveness

**DOI:** 10.1057/s41599-022-01343-w

**Published:** 2022-09-24

**Authors:** Muhamad Rosyid Jazuli, Maimanah Mohammed Idris, Penlope Yaguma

**Affiliations:** 1 Department of Science, Technology, Engineering, and Public Policy (STEaPP), University College London, London, UK; 2 Paramadina Public Policy Institute (PPPI), Universitas Paramadina, Jakarta, Indonesia

**Keywords:** Social policy, Development studies

## Abstract

Institutional quality is significantly relevant in determining national competitiveness when reflected in economic growth and successful development. Quality institutions manifest in robust ‘rules of the game’ reflected by sound governance and policymaking in at least four streams: social, economic, administrative, and political. Policy reforms intended to improve national competitiveness should aim to strengthen the streams simultaneously, whereas partial reforms could instead lead to poorer outcomes. Through the lens of institutional quality analysis, this paper critically reviews the relevance of Indonesia’s Job Creation Law, colloquially known as the Omnibus Law, to improve the country’s national competitiveness as conceptually intended. Declared as an overarching regulatory framework, the Law postulates Indonesia as prospecting for increased foreign investments. However, the Law is a partial policy reform as it overlooks the pivotal aspects necessary to improve institutional quality in Indonesia, such as inter-community relations, intellectual property regime certainty, quality vocational education, and meritocratic political recruitment. Thus, regardless of the opportunities, the Law may bring about, it may weaken national competitiveness instead. The Law is currently ruled conditionally unconstitutional by Indonesia’s Constitutional Court. If it is to be defended by the government, however, further multi-sectoral collaboration is necessary for the future implementation of the Law to enhance Indonesia’s institutional quality. In a more global context, this review indicates how developing nations should be mindful of various non-economic aspects (e.g., cultural and educational levels of the population) when undertaking policy reforms especially to enhance their national competitiveness. There may be future downsides to these implications and as it is too early to critically assess them, there is an opportunity through time and future research to do so.

## Introduction

To advance their economies, governments have increasingly realised that they need to improve their national competitiveness, often reflected in the consistent increase in their income and economic growth (Acemoglu et al., 2004; C. Lee, 2010; Rodrik et al., 2004). This paper interprets national competitiveness as a state of a country that is reflected upon its sustainable economic progress and is significantly shaped by how well the countries establish and strengthen their institutional quality (Madni, 2019; Škare et al., 2021). Empirical evidence shows that institutional quality is determined by various indicators that could be grouped in at least one of the four streams: social, economic, administrative, and political (Burns, 2007; Madni, 2019; Mamoon and Murshed, 2017; Rodrik et al., 2004). This paper attempts to explain the relevance of institutional quality to national competitiveness by critically reviewing a recent policy case in Indonesia, the Job Creation Law, commonly called the Omnibus Law (from hereon also mentioned as ‘the Law’).

At the beginning of October 2020, the government of Indonesia (GOI) passed the Omnibus Law to pursue national competitiveness (Kemenko Perekonomian, 2020b; Ninditya, 2020). The administration argues that the Law is to reform investment-related regulatory regimes targeted at attracting foreign investments. Completed within less than a year, from formulation to enactment, the Law attracted praise and criticism. Some consider it an urgent public policy for Indonesia’s economic development, especially during the Covid-19 pandemic (Kristiyanto, 2020; Suriadinata, 2019). However, others argue that the Law would complicate the country’s investment climate (Patunru and Surianta, 2020). The Law’s dynamic trajectory has recently wound up with a judicial review resulting in a court decision that is considered conditionally unconstitutional, providing the government with two years to revise it (Tresna, 2021). Such complexity prompts the question: How relevant is the Omnibus Law in pursuing Indonesia’s national competitiveness?

Attempting to address the enquiry, this paper’s objective and the main contribution to the literature are twofold. First, it explores the perceived determinants of institutional quality and its relevance to national competitiveness. Second, it appraises the policy problem regarding the Law by employing the institutional quality framework as the lens of analysis. Many have analysed the Law from a legal perspective (e.g., Riswandi, 2021; Sadono and Rahmiaji, 2021; Wole et al., 2021) or a policy innovation intended to increase investment flows (Ansari, 2020; Kristiyanto, 2020; Suriadinata, 2019). However, little has been done yet regarding the context of the Law as a public policy in pursuit of national competitiveness (as this is the GOI’s main argument (Kemenko Perekonomian, 2020b; Ninditya, 2020)) using the lens of institutional quality, hence this paper’s main contribution.

Following this introductory section is a brief review of the existing literature, which attempts to propose key perceived aspects and ‘streams’ of institutional quality considered relevant to national competitiveness. The “Methodology” section then explains the use of the critical narrative review strategy, and why it is considered suitable for exploring recent policy reforms. An explanation of the development of Indonesia’s Omnibus Law is presented in the following section. Next, the policy appraisal section endeavours to audit the Law using this lens of institutional quality. Finally, the “Discussion” and “Conclusion” sections are presented.

## Relevance of institutional quality to national competitiveness

Scholars generally agree that institutions are crucial to establishing a competitive and sustainable economy (Acemoglu et al., 2004; Addison and Baliamoune-Lutz, 2006; C. Lee, 2010; Škare et al., 2021). For example, Madni (2019, p. 581), argues that strong institutions help to “excel the living standard of economic agents.” In this case, ‘institutions’ as a term refers to North’s (1991, p. 97) definition—an often-cited one (C. Lee, 2010), that they are “humanly devised constraints that structure political, economic, and social interaction. They consist of both informal constraints (sanctions, taboos, customs, traditions, and codes of conduct), and formal rules (constitutions, laws, property rights)” (North, 1991). Institutions manifest in ‘rules of the game’—as opposed to institutions as organisations, which can be social such as religious, inter-racial, and inter-cultural interactions; political such as parliamentary and presidency; and economic such as property rights and investment policy regimes (Hill and Fee, 1995; C. Lee, 2010).

The quality of such institutions significantly determines a country’s success in economic growth and development, which is frequently considered the reflection of competitiveness (C. Lee, 2010; Mamoon and Murshed, 2017; Rodrik et al., 2004; Škare et al., 2021). However, there has been no clear consensus regarding the elements contributing to (and weakening) institutional quality. Kaufmann et al. (2003) argue that strong institutions would have robust and sound governance and policymaking ramifications. At least six aggregate indicators determine them: the rule of law, political stability, regulatory quality, government effectiveness, voice and accountability, and control of corruption. Based on the work of previous scholars (e.g., North, 1991; Rodrik et al., 2004; Williamson, 2000; Buitrago R. and Barbosa Camargo, 2021) take into consideration more indicators that are influential on institutional quality. They include trade openness, education quality, labour quality, ethnic index, intellectual property rights, and quality of infrastructure.

The above factors have been empirically analysed. Madni (2019) probes the influence of ethnic harmony, education quality, income distribution, government spending, and trade policy against institutional quality. Furthermore, Mamoon and Murshed (2017) stress the importance of quality human capital in establishing institutional quality. Lim et al. (2018) and C. Lee (2010) conclude that developing quality human capital, mainly through quality education, especially the vocational kind, is vital to establishing a superior institutional arrangement to achieve economic prosperity.

Much the same as institutions, national competitiveness is indeed diversely defined. Some understand it as the underlying driver of a nation’s prosperity (C. Lee, 2010; Škare et al., 2021). Another definition is that a nation’s competitiveness includes all attempts to actualise its global market share (Hanafi et al., 2017). Meanwhile, S. K. Lee et al. (2008) argue that a nation’s competitiveness strongly relates to the stages of its economic development, whether such level is based on factors of production, investment, or innovation and knowledge. Competitive nations run and develop their economy based on what fits their current stage of development.^1^ Accelerations within a developmental stage are possible, but skipping a step or partially undertaking the acceleration would result in poor competitiveness. A similar explanation by Addison and Baliamoune-Lutz (2006) stipulates those partial reforms intended to uplift national competitiveness result in poorer outcomes, as the promised growth and development do not materialise.^2^

National competitiveness is highlighted by sustainable economic progress with the support of other factors such as infrastructure development; efficient absorption and adaptation of advanced technologies; a stable social and political environment, and a high commitment from the government and society to the development of competent human resources (Jomo, 2006; S. K. Lee et al., 2008). A similar explanation argues that such competitiveness depends on the certainty of regulation, efficient and reliable infrastructure, and quality—including the level of literacy in human resources (Addison and Baliamoune-Lutz, 2006; Samirin et al., 2014).

The above discussion indicates that institutional quality plays a significant role in improving or weakening national competitiveness. Strong institutions have more influence over nations’ geographical locations in promoting growth (Rodrik et al., 2004). The notion that solid institutions trump geographical advantage resonates with many instances where nations with abundant natural resources and strategic geographical locations could not prosper, thanks to their poor institutional management. Rodrik et al. (2004) explain that institutional quality manifests in assurance, with or without documented regulations, and that the ‘rule of the game’ for economic matters is especially upheld and guarded.

**Fig. 1:**
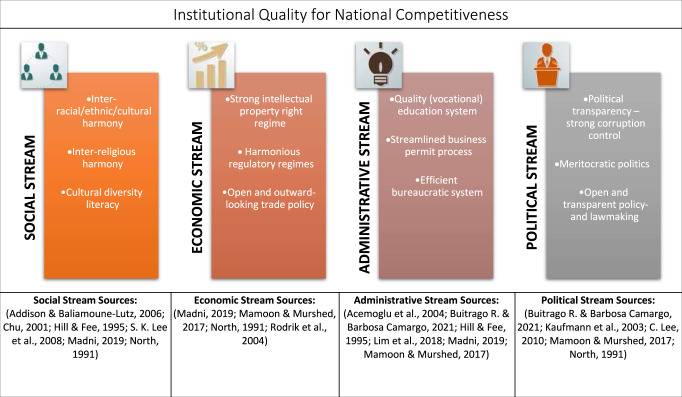
Institutional quality streams. A breakdown of social, economic, administrative and political streams for national competitiveness.

Figure 1 summarises how institutional quality contributes to strengthening national competitiveness based on the literature discussion above, and that institutional quality is built upon several qualities. These could be grouped into at least four streams, i.e., social, political, economic, and administrative. Each category contains qualities discussed and empirically tested as influential toward national competitiveness through collaborative work across different factors (Buitrago R. and Barbosa Camargo, 2021; Hill and Fee, 1995; C. Lee, 2010). It is imperative to note that institutional quality as a concept is fuzzy, meaning that it is a significantly dynamic arrangement (Addison and Baliamoune-Lutz, 2006; Buitrago R. and Barbosa Camargo, 2021). Factors influencing institutional quality are complex and interplay dynamically and contextually.^3^ To this end, although not prescriptive, the streams arguably help better structure the complexity and put it into this paper’s critical review perspective; thus, helpful for further analytical processes. Moreover, this figure emphasises that achieving national competitiveness is a multi-factor endeavour requiring multi-stakeholder collaboration (Mamoon and Murshed, 2017; Škare et al., 2021).

## Methodology

This exploratory work presents a critical narrative review (Green et al., 2006) that gathers and synthesises the developing body of literature and knowledge on Indonesia’s Omnibus Law. Such reviews have been produced for prompt analysis and conceptualisation of new significant policy changes or reforms, when well-established studies had yet to be vastly developed, e.g. (Allen et al., 2019; Hastuti et al., 2006; Ruffini, 2020). Green et al. (2006) add that critical narrative reviews may offer evidence for decision-makers, provided that the reviews are robustly and contextually produced.

For the sources of evidence, online searches were conducted for both academic and grey literature on ‘Web of Knowledge’ and ‘Google Scholar’ websites. Other relevant sources of information supplemented this.^4^ Using desk research, based on the evidence above, this paper developed foundational knowledge, reflected in Fig. 1, for further policy appraisal. This method helps develop a theoretical framework (van Thiel, 2014) that consists of perceived vital elements of institutional quality influencing national competitiveness. This framework is then employed to undertake the policy audit or appraisal. It emphasises that the Omnibus Law is appraised as a policy, referring to the government’s course of actions and decisions (Scott and Baehler, 2010; Young, 2005), instead of a legal document.

In policymaking, critical narrative reviews strongly resonate with critical policy studies or reviews that endeavour to generate perspectives on a recent policy change based upon discourses from previously relevant published research and information (Hyatt, 2013). Such reviews arguably help analyse a policy case operating in complex, dilemmatic politics, positivist perspectives often struggle to comprehend (Mulderrig et al., 2019).

## Indonesia’s omnibus Law: an overarching regulatory framework for national competitiveness

With a population of over 270 million, Indonesia is the fourth most populous country and the third-largest democracy in the world. It is also the largest archipelago nation, with more than 13,000 islands and over 500 ethnic groups (BPS, 2021). Indonesia’s GDP has recently (2021) reached US$1.158 trillion, with a per capita of US$4256 (IMF, 2021). The country is home to just under half of the Southeast Asian Countries’ or ASEAN’s population, land, and economic capacity.^5^ During the arguably authoritarian New Order Era (1967–1998), Indonesia enjoyed 7% growth annually on average. This trend ceased during the 1998 Asian Financial Crisis (World Bank, 2021). Indonesia then entered a new democratic era colloquially called *Reformasi* (Reformation). Administrations in this era have endeavoured to achieve the growth level of the New Order era, but none have been successful. Eager to push the country to achieve such an economic goal, Indonesia’s current President Joko ‘Jokowi’ Widodo argued that the country needed an overarching regulatory reform. It has now been formalised as the Job Creation Law, or better known as the Omnibus Law (Kemenko Perekonomian, 2020b; Kristiyanto, 2020).

The Omnibus Law became a public discussion topic after President Joko Widodo delivered it in his second presidential (2019–2024) inauguration speech (Kemenko Perekonomian RI, 2020b; Nurhanisah and Naufal, 2020).^6^ This term mainly referred to the Draft Law (Bill) of *Cipta Kerja* or *Ciptaker* (Job Creation), also known as the Job Creation Omnibus Bill, which proposed to simplify regulations that are considered lengthy and a hindrance to Indonesia’s economic development (Suriadinata, 2019; Wole et al., 2021). The omnibus approach reflects that decision-makers and policymakers do not want to be ‘held hostage’ by various regulations considered non-contextual. These are rules enacted in the past, without regular review, and are considered to impede the necessary policy reform process in the context of increasingly changing globalisation (Bierschbach, 2017; Garner, 2004).

The GOI signed the Bill into law (as Law 11/2020) on 5 October 2020. Drafted over a few months, the Law covers 11 regulatory clusters, namely (1) simplification of land licensing; (2) investment requirements; (3) employment; (4) ease and protection of micro, small, and medium enterprises (MSMEs); (5) ease of business; (6) research and innovation support; (7) government administration; (8) imposition of sanctions; (9) land control; (10) ease of government projects; and (11) Special Economic Zone (Kemenko Perekonomian RI, 2020a; Undang-Undang Tentang Cipta Kerja, 2020). The Law would amend more than 1200 articles in 79 laws considered less friendly to businesses and investments in Indonesia (Patunru and Surianta, 2020).

The GOI argues that efforts to develop the country towards an advanced economy must be accelerated, and the country’s national competitiveness is vital in attracting investments (Kemenko Perekonomian RI, 2020b). Theoretically and empirically, this investment flow is considered crucial for economic expansion in a country where industrialisation is still in its infancy (S. K. Lee et al., 2008; Samirin et al., 2014). The government emphasises that the investment challenge in Indonesia lies in hyper-regulation, which causes low investment realisation (Kemenko Perekonomian RI, 2020b; Mayasari, 2020). Simplifying regulations through the Law is anticipated to increase competitiveness so that investment realisation can improve and provide more job opportunities (Puspita, 2020).

**Fig. 2:**
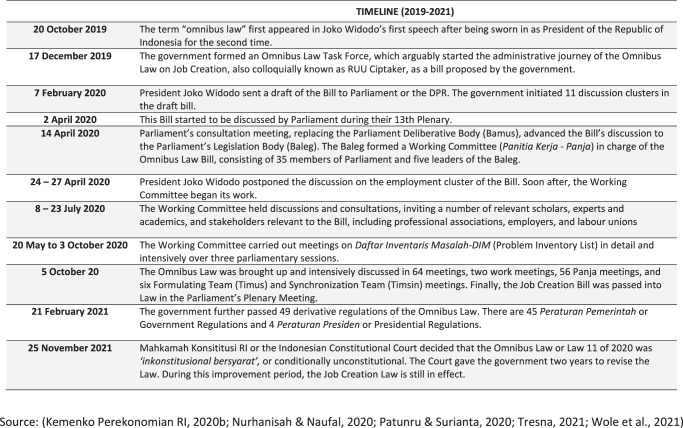
Summary of the timeline of Indonesia’s Omnibus Law. A timeline of events for the development of the Law between October 2019 and November 2021.

In February 2021, the GOI further passed 49 derivative regulations of the Omnibus Law. There are 45 *Peraturan Pemerintah* or Government Regulations and 4 *Peraturan Presiden* or Presidential Regulations. In general, the eleven clusters of the Law have been covered by these regulations. These regulations are also considered necessary for national economic recovery, especially considering Indonesia has been hit by the Covid-19 pandemic since March 2020 (Librianty, 2021). However, in November 2021, Indonesia’s Constitutional Court (*Mahkamah Konstitusi*—MK) decided that the Law was conditionally unconstitutional (*inkonstitusional bersyarat*). The Court offered additional time for the government to revise the Law within two years, during which time the Law is still in effect (Tresna, 2021). Figure 2 summarises the Law’s timeline of events from its inception to its latest changes.

## Policy appraisal of the Omnibus Law

The Omnibus Law has so far engaged the attention of policymakers, academics, and practitioners (Lie, 2020; Mayasari, 2020). Some authors are optimistic that the Law is urgent and would help Indonesia attract more foreign investment, thus promising growth, e.g. (Alaydrus, 2020; Ansari, 2020; Kristiyanto, 2020; Mayasari, 2020; Permana, 2020; Suriadinata, 2019; Wole et al., 2021). However, other researchers believe that the Law will become an additional regulation with no promising impacts on investments and may even complicate the investment regulatory regime, e.g. (Florentin, 2020; Huzaini, 2020; Jazuli, 2021; Patunru and Surianta, 2020; Prasasti, 2020). Further, the implementation and outcomes of the Law are still uncertain due to the poor consultations with stakeholders such as trade unions, civil society, and academia (OECD, 2020). Based on these debates, the following section appraises the Law through the lens of the institutional quality framework presented in Fig. 1. The policy appraisal aims to explain how the Law may operate within the different institutional quality streams: social, economic, administrative, and political.

### Social stream: operating in an intense inter-ethnic atmosphere

Soon after the passing of the Job Creation Law, the GOI initiated a social security programme for formal workers, namely the Job Loss Insurance (*Jaminan Kehilangan Pekerjaan*—JKP) (Pratama, 2020). This programme is championed as the government’s social responsibility to workers to encourage them to return to the workforce more successfully at the time or as soon as they get laid off (Aditiasari, 2020). Workers receiving support through this programme will have access to job market information, training, and cash guarantees (Arieza, 2020). Through a social safety net like this, Indonesia’s Omnibus Law may contribute to maintaining labour market stability, supporting Indonesia’s efforts to continue improving and growing its industrialisation.

However, this social safety shows that the potential for layoffs is instead more apparent in the era of the Job Creation Law. The Law arguably gives employers the authority to employ their workers on a contractual basis for an indefinite period (Arnani, 2020; Sadono and Rahmiaji, 2021). While the GOI argues that the regulation mandates employers to give proper layoff compensations (Kemenko Perekonomian RI, 2020b), the Law is still widely believed to disproportionately benefit capital owners over other actors (Florentin, 2020; Sadono and Rahmiaji, 2021). In the Indonesian context, when engaging these capital owners, it is crucial to understand the historical (yet informal) and strong cultural and ethnic sentiments which are still occurrent.

The capital owners are dominated by persons of Chinese descent and non-Muslims (Chua, 2006; Koning, 2007)—as opposed to the Indigenous (*the pribumis*) and Muslim majority. Some have argued that Indonesia has partly been spared from horizontal conflicts, involving inter-ethnic or inter-community disputes (Aziz, 2014; Subchi and Alkaf, 2018). However, since the last two presidential elections, inter-ethnic frictions often rose when Indonesians of Chinese descent were accused of siding with one of the two presidential contenders, although these disagreements rarely led to physical conflicts (Pertiwi, 2021). Such a societal dynamic puts significant pressure on economic and market stability in the country, which to some experts, endangers investment realisations (Chu, 2001; Madni, 2019).

Others argue that Indonesia’s inter-ethnic and inter-religious relations are indeed fragile (Atmaja and Fachrurazi, 2018; Nagata, 2003). In its mid-term development plan, the government confirmed these aspects of fragility due to the declining trend of religious moderation for tolerance and harmony among different groups, especially those of different ethnicities and religions (Bappenas, 2020). These concerns are also indicated by the decreasing score of the ‘Religious Harmony Index’, produced by Indonesia’s Ministry of Religious Affairs. It shows that the level of tolerance, equality, and cooperation among religious believers has been dynamic but has decreased from 75.4 in 2015 to 73.8 in 2019 (Bappenas, 2020).^7^

All this indicates that navigating these inter-group relations and tensions is key to policy success. In drafting the Omnibus Law, the government maintains that they actively consulted various civil society groups (Nurhanisah and Naufal, 2020). However, they did not involve major organisations, such as Nahdlatul Ulama (Revival of the Muslim Scholars), Muhammadiyah (the Followers of Muhammad), and Majelis Ulama Indonesia (Indonesia’s Muslim Scholar Council). Regarding the complex Indonesian policymaking context, these Islamic groups hold significant powerful political influence (Jazuli et al., 2021; Wahyu, 2015). Consequently, their reservations toward the Law arose soon after it was enacted (Arnani, 2020; Sahal, 2020). To this end, rather than promoting prosperity, the Law intensifies the social problems regarding inter-group relations, leading to perpetual social instability in the country. Since the passing of the Law, the GOI has yet to engage actively with some politically influential civil society groups, especially NU and Muhammadiyah, as resistance from these groups can easily be observed (Arnani, 2020).

### Economic stream: a longing for quality regulatory regimes to support growth

Economically, the Law may serve as a strategic driver for improving various indicators of doing business in Indonesia. These include dealing with construction permits, registering property, paying taxes, and resolving bankruptcies. In this case, the Law is considered a breakthrough, an indispensable method for Indonesia to seize an opportunity to be more competitive than other developing countries (Kemenko Perekonomian RI, 2020b; Laoli, 2020). It is expected that the Law will attract investments from foreign sources, which can significantly drive up economic growth (Permana, 2020).

**Fig. 3:**
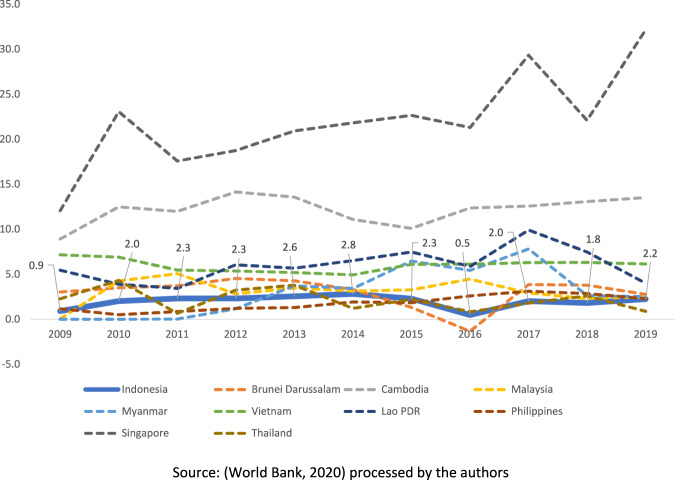
ASEAN countries’ net FDI inflows 2009–2019 (percentage of GDP). Despite being the biggest economy in the region, Indonesia’s net FDI inflows to GDP between 2009-2019 ranks relatively low compared to the other ASEAN countries.

**Fig. 4:**
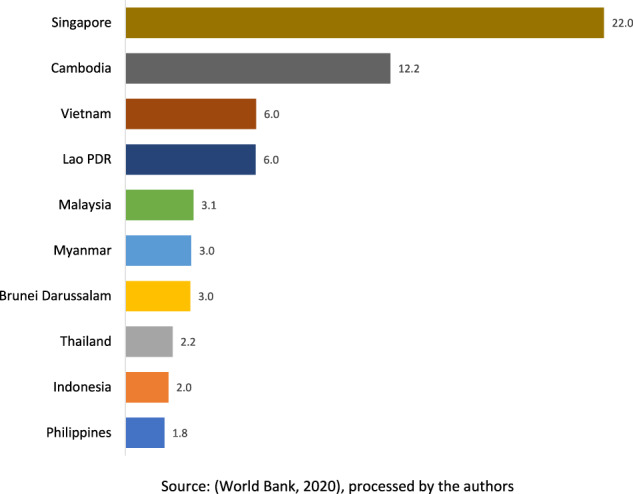
ASEAN countries’ average net FDI inflows 2009–2019 (percentage of GDP). The average contribution of FDI to Indonesia’s economy between 2009 and 2019 is at 2 percent, making it comparatively poorer to most other countries in the region.

**Fig. 5:**
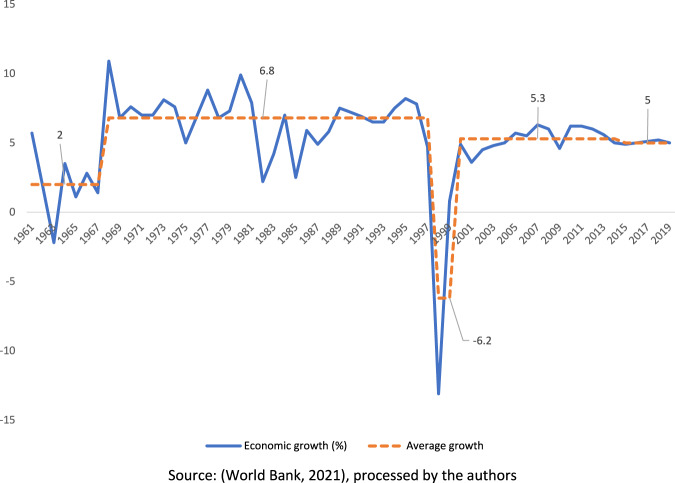
Indonesia’s economic growth record (percentage). A representation of annual economic growth of Indonesia (from 1961 to 2019) and the overall average growth.

Despite the current positive average growth of around 5%, investment contribution toward Indonesia’s GDP is in decline. Moreover, the net foreign direct investment (FDI) inflows to Indonesia reached only about 2.8% of GDP (in 2014) and decreased to 2.2% (in 2019) (see Fig. 3).^8^ Meanwhile (between 2009 and 2019), the average percentage of net FDI inflow to Indonesia’s GDP is still below that of most neighbouring ASEAN countries (see Fig. 4). Furthermore, in the current Reformation era (since 1998), Indonesia’s economic growth is relatively slower (around 5%) than in the previous New Order period (6.8%) (see Fig. 5). To this end, the Omnibus Law may help advance the country economically through potential FDI growth.

Nevertheless, uncertainty in investment regulations is a major challenge in Indonesia, leading to these poor economic outcomes (OECD, 2020; Samirin et al., 2014). Indonesia’s Intellectual Property (IP) regime is a notable example of regulatory uncertainty. Since 1998, administrations have attempted to reform Indonesia’s IP regime to meet international expectations (Bappenas, 2016; Butt and Lindsey, 2009). This endeavour arguably contributed to bringing Indonesia in line with the World Trade Organisation’s Trade-Related Aspects of Intellectual Property Rights (TRIPs) Agreement (Butt and Lindsey, 2009; Susanti et al., 2019). However, the IP regulations have rarely been enforced, hence IP infringements have persisted regardless of the reforms (Kusumadara, 2000; OECD, 2020; Susanti et al., 2019). For instance, in 2019, the Indonesian film industry incurred losses of approximately Rp1.4 trillion (around US$100 million) due to piracy and illegal downloads (Anggoro, 2019).^9^

Several scholars have critiqued the Omnibus Law as undermining instead of strengthening the IP regime. For instance, the Law is considered to discourage the national patent system, allowing those with international patents to do business in the country. While it may look like it is creating an easier way of doing business, the Law could lower the local economy’s competitiveness, leaving small business players, mainly MSMEs, fighting the big ones without necessary protection (Riswandi, 2021). Instead of improving and enhancing the enforcement of the existing IP regime, the Law attempts to cancel key rules, especially concerning patents (Ashar, 2020; Saidin, 2020).

Besides, several laws have in fact existed to regulate development planning and budgeting intended to help Indonesia develop and grow effectively. These rules include Law 17/2003 on State Finances, Law 25/2004 on the National Development Planning System, and Law 23/2004 on Regional Government. These laws have fundamentally different philosophical underpinnings that give rise to “disconnections of planning and budgeting” (Wasono and Maulana, 2018, p. 14). The State Finances Law, for example, mandates a significant observance of programmes and performances as the basis for budgeting, while the National Planning Law prefers departmental functions. Moreover, the State Finances Law also mandates the implementation of performance-based budgeting. Sitepu et al. (2014) found that up until 2014, no such practices had been sighted, as most relevant state officials disagreed that making performance the basis of budgeting could encourage budgeting efficiency.

Indonesia’s experience with such laws—although promising potential growth, poses a worrying question on the effectiveness of the Job Creation Law in helping the country’s economy advance. The Law may arguably only complicate the regulatory ecosystem, discouraging rather than attracting investors (Jazuli, 2021; Patunru and Surianta, 2020).

### Administrative stream: facing insufficient HR and scattered business permit process

Since 2001, more than 500 local governments in Indonesia have administratively, been deemed autonomous and can pass any regulation, especially when obtaining business and investment permits (OECD, 2020; Wahyuni and Ng, 2012). Unfortunately, each local government’s policies frequently conflict with central and other provincial governments (World Bank, 2019). Such a conflictual regulatory regime has become a national concern, making it difficult for investments to enter the country (Bappenas, 2020). The presence of the Omnibus Law and its centralised approach means that the business and investment licensing process is simpler and faster. This legal framework would, in due course, form and implement a licensing system through the ‘Online Single Submission’ (OSS) system—considered attractive to investors (Finaka and Nurhanisah, 2020).

Besides, the government argued that the Law is necessary due to the Covid-19 pandemic, which slowed the economy by 5.32% in the second quarter of 2020 (BPS, 2020a). Due to the pandemic (from 2020), unemployment is predicted to soar to more than 12 million over the next few years, which would intensify poverty in the country (Zuraya, 2020). To this end, the Omnibus Law is expected to improve Indonesia’s business climate, tapping into more foreign investments and expanding the job market (Kemenko Perekonomian RI, 2020b; Permana, 2020).

Nevertheless, regardless of the job expansion potential, Indonesia’s incompetent labour force is a severe issue for the country. Out of its massive labour force, amounting to around 138 million, the number of competent or skilled workers in their fields by 2020 only reached about 40 million (33%) (BPS, 2020b). There are about 70 million junior high school graduates (*Sekolah Menengah Pertama*—SMP) or below (see Table 1). This number corresponds to those who became informal workers, reaching around 70 million people (BPS, 2020b; Jayani, 2020). Indonesia’s vast low-educated, and informal workforce correlates with workers with limited skills and capabilities and produces less competitive products (Putra, 2019). In contrast, national competitiveness in the early phases of economic development largely depends on skilled human resources and a high level of educational qualifications (S. K. Lee et al., 2008).

**Table 1:** Educational backgrounds of Indonesia’s labour force (as of February 2020).

Education	Employed	Unemployed	Total
No/never not been to school	3,292,479	34,779	3,327,258
No/have not finished elementary school	15,022,932	341,180	15,364,112
Primary School	32,636,991	1,003,901	33,640,892
Junior High School	23,490,599	1,242,248	24,732,847
High School (General)	24,026,896	1,743,696	25,770,592
High School (Vocational)	15,482,001	1,435,589	16,917,590
Diploma I/II/III/Academy	3,661,481	265,400	3,926,881
University	13,410,429	815,407	14,225,836
Total	131,023,808	6,882,200	137,906,008

Source: BPS (2020b).

**Fig. 6:**
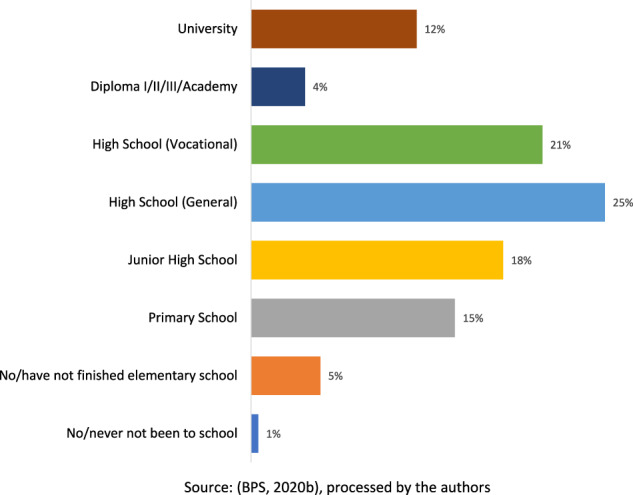
Percentage of the unemployed by education. A breakdown of unemployment rates in Indonesia based on different educational levels in February 2020.

Since 2014, the GOI has tried to solve the skills gap by establishing as many vocational high schools or *sekolah menangah kejuruan* (SMK) as possible. This aims to produce vocational graduates expected to meet the needs of Indonesian industries for skilled workers (Idris, 2019). However, these existing labour-intensive industries do not optimally absorb vocational graduates (see Fig. 6). This is because national vocational education is deemed irrelevant to the workforce demands of the existing industries, especially the labour-intensive types (Movanita, 2019). In addition, students entering vocational schools are generally not the most talented, primarily due to the perception in society that vocational schools are second-rate, inferior schools (Dardiri, 2012). In the end, as of 2020, vocational schooling has become a significant contributor to the unemployment rate in Indonesia (BPS, 2020b).

To this end, the Omnibus Law may serve as a powerful administrative tool to improve Indonesia’s business climate. However, the above labour dynamic indicates that, unfortunately, there is a significant gap in the supply of quality workers, which the Omnibus Law has yet to address. Therefore, any massive job openings promised by the Law would be irrelevant because the human capital’s readiness is unsatisfactory.

### Political stream: corruption putting law-making at stake

Alongside its centralised approach, the Omnibus Law is anticipated to significantly reduce the long process of obtaining permits and suppress illegal levies and corrupt practices, especially related to business and investment licensing (Finaka and Nurhanisah, 2020; Laeis, 2020). The central government will have the authority to intervene in setting local tax rates and regional levies and supervising relevant regional regulations, which many consider a significant obstacle to investment. The Law, to this end, is considered indispensable for Indonesia, as it allows it to seize an opportunity to be more competitive than other developing countries (Laoli, 2020). This way, the Law would contribute significantly to Indonesia’s anti-corruption agenda.

**Fig. 7:**
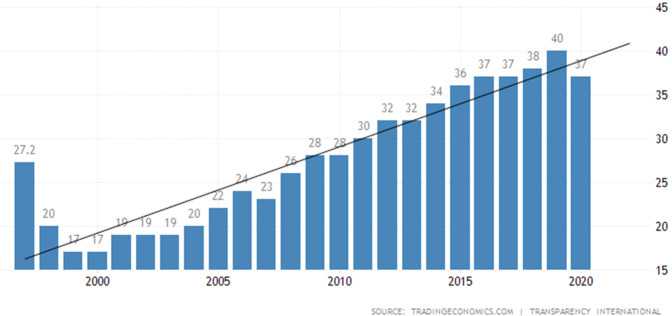
Indonesia’s Corruption Perception Index (CPI) trajectory. A 23-year trend of Indonesia’s CPI showing a steady progression from 1997 to 2020.

Since 1998, various administrations have prioritised an anti-corruption policy to pursue transparent and accountable governance (Umam and Head, 2020). The positive impacts of such an endeavour can be seen in the positive trend of the country’s Corruption Perception Index (CPI) score (see Fig. 7). This Index is often used as an anti-corruption regime measurement (Kaufmann et al., 2003; Umam and Head, 2020). But this trajectory is plateauing and is predicted to decline. Umam and Head (2020) argue that there have been threats posed by resilient networks of politically co-opted businesses reconsolidating and resisting anti-corruption measures.

Moreover, Indonesia still ranks low in terms of CPI, in most cases, lower than 100 out of 170 countries (Transparency International, 2021). One could argue that the index has nothing to do with investment as countries like Vietnam, which enjoyed better investment to GDP rates, rank lower than Indonesia. However, compared to countries like Singapore, which consistently rank better and have a higher investment-to-GDP ratio, the index finds some credibility (Quah, 2021). Samirin et al. (2014) explain that, in the end, the anti-corruption rank is a strong signal of policy certainty and, therefore a significant driver in attracting and realising investments. Indeed, investors and businesspeople rely much more on how the non-corrupt administrations could signal to the market; that there will be a level playing field for both business players and investors (Rodrik et al., 2004; Škare et al., 2021). Such signals can be provided when capable people oversee the departments where investments are managed (K. Y. Lee, 2000).

Such a meritocratic political system significantly influences the placement of these capable officials (D. S. Lee and Schuler, 2020). Empirically, some politically autocratic nations, like the East and Southeast-Asian countries, including Korea, Indonesia (before 1998), and Singapore, tend to favour technocratically shrewd people over the politically able. They allowed them to chair ministries or departments, especially those with crucial portfolios. In Singapore, for instance, capable people also put more effort into establishing a robust anti-corruption regime with high accountability. Singapore’s continuously refined meritocratic political recruitment attracts and retains the best people to become top political officials and civil servants, allowing the country to produce incorruptible and effective policies (Bellows, 2009; K. Y. Lee, 2000).

However, in Indonesia (after 1998), political recruitment has been far from procedural, let alone meritocratic (Junaidi, 2020). After 1998, political parties consistently failed to field their cadres during national and regional elections. Indeed, this also showed the subsequent poor political recruitment and leadership choices. As a corollary to that situation, the government lacks capable policy, decision-makers, and shrewd legislative lawmakers. At constant stake are the quality of laws and regulations and the quality of the anti-corruption regime (Umam and Head, 2020). Related to this concern, some argue that the making of the Omnibus Law was in itself corrupt (Sadono and Rahmiaji, 2021; TI Indonesia, 2020).

According to Transparency International Indonesia (2020), the fast-paced ratification of the Law is considered improper for such an overarching regulation, ignoring the principle of policymaking deliberation and damaging public aspirations. This view ultimately reflects the low quality of lawmakers, namely top officials in the administration and representatives of political parties in Parliament (Anggraeni, 2020). The culmination of this debate was arguably the Indonesian Constitutional Court’s decision that the Law is considered conditionally unconstitutional, and the government was given two years to revise it (Tresna, 2021).

## Discussion

The policy appraisal above indicates that, if implemented effectively, the Law could improve the country’s economic advancement. It also serves as a legal foundation for matters that involve Indonesia’s competitiveness. These are matters such as, protecting workers from job loss, attracting FDI’s, simplifying the business permit process, and suppressing illegal business and investment levies. Nevertheless, the Law operates in a policy setting that is socially, economically, administratively, and politically complex and risky. It has been received with mixed opinions that include doubts about its effectiveness and relevance to improving the country’s competitiveness. There have been underpinning factors, that may lessen its impact, including, the nation’s fragile social fabric, infamous conflictual regulatory atmosphere, and incompetent political nature. These factors have arguably led to the latest judicial review, resulting in ‘the Law is conditionally unconstitutional’.

This brief yet dynamic backdrop to the Law warrants the previous enquiry into the relevance of the Omnibus Law to Indonesia’s competitiveness. The aforementioned studies have found that institutional quality is vital to strengthening national competitiveness, often reflected by economic expansion and growth (Acemoglu et al., 2004; C. Lee, 2010; Rodrik et al., 2004; Škare et al., 2021). Such institutional quality manifests itself in streams of sub-qualities, four of which are used in this paper: social, economic, administrative, and political.

As a policy intended to boost Indonesia’s national competitiveness, the Omnibus Law can be used within a dynamic policy environment instead of in a policy vacuum. Using the multi-stream institutional quality framework, the Law (as a policy) is a promising tool to aid Indonesia’s economic expansion. It ensures a more centralised process when it comes to realising investments. It will indeed help Indonesia be more open to international trade and entice more investments if it is well-implemented. However, this paper’s appraisal indicates that the Law could be inconsequential to the intended economic growth.

Addison and Baliamoune-Lutz (2006) and S. K. Lee et al. (2008) warn that, eventually, partial economic reforms will result in poor outcomes such as slower unsustainable growth. Per this paper’s appraisal, the Omnibus Law is one such partial policy reform in pursuing national competitiveness. The following propositions discuss why, instead of strengthening institutional quality—thus improving Indonesia’s national competitiveness, the Law may undermine it and lead to unsustainable growth.

Firstly, the Law’s ability to boost stability in vital areas such as social and political movements is limited. Claimed by the government as an overarching policy, the Law seems insensitive toward non-economic sentiments such as ethnic and religious prejudices deeply rooted, if not innate, in Indonesia’s multi-cultural communities. While in the past, there have been consultations with non-state actors for policy success, engagement with religious and civil society leaders was limited during the policymaking process of the Law. This has resulted in demonstrations and constant criticisms of the Law.^10^ To this end, Chu (2001) and Madni (2019) could not be more right for probing intercultural relationships for institutional quality measurement and finding it a significant determining factor.

Secondly, the Omnibus Law omits a fundamental aspect of an investment-friendly environment: the protection of IP for the local economy. Meanwhile, empirically, such protection holds considerable significance in strengthening nations’ institutional quality, thus improving their competitiveness. Instead of establishing and further strengthening policy certainty in the investment environment (with all the public reservations and debates), the Law seems to undermine it. While the government argues that the Law is implemented to loosen up the IP regime, believing that such a condition is needed to attract investment, experts and empirical evidence demonstrate otherwise (Acemoglu et al., 2004; Samirin et al., 2014).

Thirdly, evidence shows that laws in Indonesia take up an inconceivable long time, if indefinite, to implement. The lack of quality in human resources is hardly considered by the Law, whereas evidence shows that a well-resourced, alongside quality vocational education, plays an essential role in improving national competitiveness (C. Lee, 2010; Lim et al., 2018; Madni, 2019). One may argue that there is no need for the Law to manage the human resources issue as it is already under the authority of the Ministry of Education. However, as per this appraisal, such a perspective would only lead to a partial reform which empirically has not led to improvement. Ultimately, the Law has stirred up debates, weakened national competitiveness, and may even discourage instead of attracting investments.

Lastly and crucially, when flagging up Indonesia’s development progress, one finds that the recent average growth is in a state of decline. The government argues that the Omnibus Law will be the panacea of such a growth pursuit. It promises policy certainty to attract more investments (Ninditya, 2020; Nurhanisah and Naufal, 2020). However, the Law has produced continuous debates and uncertainty that have led to the Court’s decision that the Law is conditionally unconstitutional. Unfortunately, such development of the Law sends contrasting signals to investment markets. It indicates that instead of the Rule of Law and a level playing field for investments to be realised, administrative and political red tapes are and will remain prevalent in the country. To a more global extent, such a policy dynamic suggests that policy endeavours to advance the national competitiveness of countries, especially the Global South nations, should be considerate of the vast foundational, non-economic aspects such as the people’s cultural and educational levels.

In the end, as a reform policy, the Omnibus Law brings opportunities and risks (OECD, 2020). The policy’s centralised approach would potentially (if effectively implemented) address the sometimes unclear and uncoordinated roles and responsibilities of Indonesian ministries and agencies, especially those related to the investment regime in the country. However, the expectedly effective implementation would need a well-crafted policy design. It would be an enormous task requiring consistent and far-reaching communications, collaborations, and consultations with multi-sector non-governmental interlocutors, both individual and institutional. This wide-ranging engagement with diverse stakeholders is not to undermine the need to act decisively and swiftly to implement the Law, but instead to ensure that the policy design is effective based upon constructive dialogues and the Indonesian people’s trust and cooperation. These relevant stakeholders may include but are not limited to civil society communities—which in the Indonesian context indeed involve the religious organisations mentioned previously, academia (universities, research centres, think tanks, etc.), various political parties, trade unions and business associations.

## Conclusion

This critical review has narratively demonstrated that sustainable economic growth as the main reflection of national competitiveness requires support from strong institutions manifested in at least four streams: social, economic, administrative, and political. Undertaking partial measures to establish such institutional quality, for example, focusing only on one or two streams, eventually undermines the pursuit of national competitiveness. Thus, a more comprehensive policy approach involving improvements in all institutional quality streams is recommended; instead of accelerating one stream over another, as the Indonesian Omnibus Law is intended.

Through the Law, the GOI has signalled its commitment to attracting investments to boost national competitiveness, sending a message to investment markets of favourable relevant regulatory regimes that are less stifling. However, through the institutional quality lens, this paper’s analysis shows that the Law would, in the end, serve as partial policy reform. The GOI misses other vital aspects such as strengthening inter-community relations, ensuring IP regime certainty, improving quality vocational education, and establishing meritocratic political recruitment and regeneration. The most recent development, where the Law has been considered conditionally unconstitutional only emphasises that the policy is irrelevant to the Indonesian quest for national competitiveness.

Further research on how the Law is implemented is indeed necessary. Nevertheless, this paper has argued that pursuing national competitiveness requires a variety of policy tools in different fields, which are reflected by the aforementioned institutional quality streams. To this end, if the GOI still supports the Omnibus Law as an important mechanism in improving Indonesia’s competitiveness, then the Law should prioritise multi-sectoral collaboration. That includes multi-sector stakeholders such as leaders of religious and civil society organisations, political parties, universities, and research centres.

## Data Availability

All figures, illustrations, and datasets are open-sourced and do not require copyright approval. All are referenced within this document.

## Footnotes

1 In the post-World War II era, some Asian economic ‘miracles’ underwent similar trajectories, such as Japan, South Korea, Taiwan, Hong Kong, and Singapore. S. K. Lee et al. (2008) explain that, for instance, Singapore, had managed to accelerate its industrialisation stage within around three decades from 1965 to the early 1990s. As a result, the country has a robust, modern labour force, with low unemployment rates, before advancing to attract more foreign investments focused more on the research and development sector.

2 An example of such a partial reform is installing anti-corruption and new public management values in the bureaucratic systems when people’s literacy and culture have yet to be sufficiently exposed to and immersed in such modern arrangements (Addison and Baliamoune-Lutz, 2006). Many policy reforms in developing countries arguably wound up being partial and failing because they did not have similarly contextual foundations such as the culture of openness and transparency in bureaucracy considered readier in more advanced countries (Schick, 1998).

3 In this case, papers related to institutional quality do not seem to differ much in terms of the influential, independent factors or qualities towards institutional quality. However, papers that cover in full or include partly non-Western country studies, such as Asian or African countries, tend to include social stability in the form of stable inter-community (ethnicity, religions, etc.) relations as an important factor (e.g. Madni, 2019; World Bank, 2003).

4 Grey literature refers to any information not published by or via traditional academic publishing procedures and practices. In general, this type of information source includes both published and unpublished pieces. The published ones are such as newspaper articles, government or council documents, policy briefs or papers, think tank reports, conference papers, blog posts, and speeches. Meanwhile, the unpublished are like doctoral theses. In the context of policymaking, as Lawrence (2018) argues, these sources of information are “becoming a key tool in influencing public debate and part of the evidence-based for public policy and practice.” Grey literature has played an increasingly significant role in shaping policy discourses, thus influencing decision-making for policy (Cavet and Sloper, 2004; Lawrence, 2018).

5 ASEAN stands for Association of Southeast Asian Nations. Currently, it consists of 11 countries: Brunei, Myanmar, Cambodia, Timor-Leste, Indonesia, Laos, Malaysia, the Philippines, Singapore, Thailand, and Vietnam.

6 In 2019, the Indonesian public was shocked by the vastly circulated information that from June to August 2019, around 33 companies decided to move their production centres out of China, from which 23 of them moved to Vietnam. The rest went to Cambodia, India, Malaysia, Mexico, Serbia, and Thailand, while none to Indonesia (Lidwina, 2019; Patunru and Surianta, 2020; Sidik, 2020). Many, including the administration itself, blamed it on the complicated business and investment licensing process in Indonesia (Wareza, 2019). For instance, in practice, when opening a factory, a business person must have permits from various governments at various levels, which are prone to illegal fees (Samirin et al., 2014). The administration, concerned with China’s investment decisions, arguably embarked on drafting and passing the Omnibus Law (Hartati, 2019; Sidik, 2020).

7 The Indonesian Ministry of Religious Affairs explains that the Religious Harmony Index reflects the relationship between religious communities based on how tolerant and equal they are, “in practicing religion, and working together in building the society, nation and state of the Republic of Indonesia based on Pancasila and the 1945 Constitution” (Sila and Fakhruddin, 2019). Based on that description, an annual survey has been carried out since 2016, drawing data based on three elements to be used as key indicators: tolerance, equality, and cooperation.

8 Simply put, capital or investment inflows reflect the amount of investment that enters a country. Practically, if the capital account transactions, in the balance of payments, are at a surplus level, this situation results in net investment or capital inflows or net inflows into a country. The positive figure of net investment inflows tends to strengthen foreign exchange reserves as well as the balance of payments performance of the country, and vice versa (Nizar, 2007). For a more detailed explanation, see e.g. Combes et al. (2011) and World Bank (2022).

9 Considering the exchange rate in 2019, US$1 equals approximately Rp14,000.

10 For example, in the case of fossil fuel subventions, the policy has been arguably successful and significantly supported by religious organisation leaders, due to government’s efforts for continuous consultation with them (Jazuli et al., 2021).
